# Medical Opinion and Movement

**Published:** 1907-11-09

**Authors:** 


### Medical Opinion and Movement.

No more convincing evidence could be offered of the urgent necessity for the recent Act of Parliament providing for the medical inspection of children in the elementary schools than the recent report of Dr. James Kerr, medical officer to the London County Council for education purposes. This report, in fact, carries us beyond measures of medical inspection, and opens up the question of treatment as a problem for immediate consideration by the Council if children are to have a fair chance of benefiting by the education afforded to them. Compulsory education seems necessarily to involve the obligation to provide that the child is in a physically fit condition to receive the instruction, and, as Dr. Kerr points out, it is hopeless to look to the private practitioner or the public hospital to provide adequate means of relief for the numberless cases of defective eyesight, discharging ears, carious teeth, and the like serious physical defects. If the object of State education be to insure in future generations a people capable of performing their duties as citizens of a great nation, then physical fitness is the first essential, for without it mental fitness is an impossibility. To attempt to develop the mind without making provision for the health of the body is to begin at the wrong end. All this has been said many times before, but Dr. Kerr's report demonstrates by facts what might easily have been foreseen by theory. Now, however, that we have a special medical department attached to the Board of Education, we may look for a serious consideration of this most important problem.

Dr. Frederick Taylor is to be congratulated upon the ability with which he performed the duty of Harveian Orator for the present year. His oration, entitled "The Need of Research in Medicine," will well repay perusal. He gives a brief but interesting survey of the results of laboratory research in recent years, and skilfully turns it to account to demonstrate how much there is yet to be done. In this survey bacteriology naturally takes a foremost place, but the work of the physiologists is hardly less important, and his eulogium of the brilliant researches of Professor E. Starling and Professor Pawlow is well deserved. In dealing with the question of research the orator naturally refers to the vivisection problem and the recent investigations of the Royal Commission on the subject, and this part of the oration affords one of the most concise, conclusive, and convincing justifications of modern methods of experiments on animals that have yet been made by any member of the profession. We would gladly see it reproduced in any of our lay contemporaries for the edification of the public mind and the better understanding of the people on the subject. He deals with the two main objections of the antivivisectionists—that vivisection is useless and that it is immoral, and his refutation of both is complete and overwhelming. We heartily commend it to the attention of any of the profession who may have the opportunity of enlightening public opinion on the question.

Suprarenal extract has failed to do for Addison's disease what the administration of thyroid has done for myxœdema, and we are still awaiting a specific and rational method of treatment. Dr. E. Grawitz has recently published two cases in which apparent cure has been effected. One case was that of a young man who developed the disease while carrying out his term of military service. There was considerable digestive disturbance, with severe vomiting after food. The trial breakfast showed diminished hydrochloric acid and motor insufficiency. Lavage of the stomach and the administration of hydrochloric acid was the treatment adopted, and in the course of several weeks the patient rapidly improved, put on weight, and was eventually restored to health. According to Kohn and others, the active substance of the suprarenals is contained in the so-called chromaffine cells, which also exist in the sympathetic. Destruction of the adrenal chromaffine cells is followed by a vicarious hyperplasia of similar cells in the paraganglions and sympathetic. Grawitz points out that Addison's disease in common with pernicious anæmia and severe cachexia is associated with severe gastric disturbances, which are in many cases an important factor in the rapid decline of the patient. If they can be removed by the method of treatment adopted by the author, time may be given to the system to work out its own salvation either by a recovery of the function of the suprarenals or by a hyperplastic development of the corresponding cells of the paraganglions and sympathetic system.

Psychologists have done much in recent years to remove the study of the mind out of the region of speculative philosophy, and by applying modern methods of scientific research to bring it into line with the more exact physical and physiological sciences. The complex mental processes have been analysed and their time-durations as accurately measured as the rate of a simple nerve impulse along a peripheral nerve. Considerable interest has been aroused by a new method of mental analysis, by which it is claimed the strength of an emotion can be measured by the movements of a galvanometer. Dr. F. Peterson, of Columbia University, New York, has carried out some interesting researches on the subject in conjunction with Dr. Jung at Zurich, and he gave the results of his work in a paper at the Exeter meeting of the British Medical Association. According to Dr. Peterson, different stimuli—sensory, such as a loud noise, or verbal, in the form of a question or command—will produce a deviation of the galvanometer, varying according to the degree of emotion which the stimulus calls forth. Marked differences are shown between normal and abnormal mental states. Thus in katatonia the latent period is greatly increased. The cause of these electrical changes accompanying the emotions is not quite evident, but the suggestion which receives most support is that of Yarchanoff, who looked upon these phenomena as due to a secretory current of electricity associated with the sweat glands.

